# Congenital chloride diarrhea and Pendred syndrome: case report of siblings with two rare recessive disorders of SLC26 family genes

**DOI:** 10.1186/s12881-020-01023-z

**Published:** 2020-04-15

**Authors:** Eva Lindberg, Claes Moller, Juha Kere, Satu Wedenoja, Agneta Anderzén-Carlsson

**Affiliations:** 1 Department of Paediatrics, Örebro University Hospital, SE-701 85 Orebro, Sweden; 2 Audiological Research Centre, Faculty of Medicine and Health, Örebro University, Örebro, Sweden; 3 The Swedish Institute for Disability Research, Örebro University, Örebro, Sweden; 4 Department of Biosciences and Nutrition, Karolinska Institutet, Stockholm, Sweden; 5 Stem Cells and Metabolism Research Program, University of Helsinki, and Folkhälsan Research Center, Helsinki, Finland; 6 Obstetrics and Gynecology, University of Helsinki and Helsinki University Hospital, Helsinki, Finland; 7 University Health Care Research Center, Faculty of Medicine and Health, Örebro University, Örebro, Sweden

**Keywords:** Case report, Congenital chloride diarrhea, Deafness, Neonatal diarrhea, Pendred syndrome

## Abstract

**Background:**

Congenital chloride diarrhea (CLD; OMIM 214700) is a rare autosomal recessive disorder caused by pathogenic variations in the *solute carrier family 26 member A3* (*SLC26A3*) gene. Without salt substitution, this chronic diarrheal disorder causes severe dehydration and electrolyte disturbances. Homozygous variants in the nearby gene *SLC26A4* disrupt anion exchange in the inner ear and the thyroid, causing Pendred syndrome (PDS; OMIM 274600), which is the most frequent form of syndromic deafness.

**Case presentation:**

We report an unusual co-occurrence of two rare homozygous mutations in both the *SLC26A3* and *SLC26A4* genes, causing a rare combination of both CLD and PDS in two siblings. Although the clinical pictures were typical, the combined loss of these anion transporters might modulate the risk of renal injury associated with CLD.

**Conclusions:**

Familial presentation of two rare autosomal recessive disorders with loss of function of different SLC26 anion transporters is described. Independent homozygous variants in the *SLC26A3* and *SLC26A4* genes cause CLD and PDS in siblings, shedding light on co-occurrence of rare recessive traits in the progeny of consanguineous couples.

## Background

Congenital chloride diarrhea (CLD; OMIM 214700) is a rare autosomal recessive disorder. It is caused by pathogenic variations in the *solute carrier family 26 member A3* (*SLC26A3*) gene on chromosome 7q31, which encodes for an apical epithelial chloride-bicarbonate exchanger of the terminal ileum and large intestine [1]. Homozygous mutations in the nearby gene *SLC26A4* disrupt anion exchange in the inner ear and the thyroid, causing the recessive Pendred syndrome (OMIM 274600) [2, 3].

Sporadic cases of *SLC26A3-*related CLD have been reported worldwide, but founder mutations are associated with higher incidences in Finland (1:30,000 to 1:40,000), Poland (1:200,000) and in the Arab Middle East (1:3200–1:5000) [4]. CLD is characterized by watery diarrhea with high levels of chloride (> 90 mmol/l) and low pH, leading to hypochloremia, hypokalemia, metabolic alkalosis and dehydration. During pregnancy, fetal CLD is associated with polyhydramnios and dilated intestinal loops resembling ultrasonic signs of intestinal obstruction [5, 6]. CLD newborns are often slightly premature and have distended abdomens and watery diarrhea. Although untreated dehydration and electrolyte imbalance are life-threatening, salt replacement with NaCl and KCl solutions allows favorable long-term outcome in patients with CLD [6]. CLD might be complicated by renal injury, intestinal inflammation and male subfertility [7].

*SLC26A4*-related Pendred syndrome (PDS) is characterized by hearing loss, starting in late childhood or early adulthood, and goiter with hypothyroidism [8]. The severity and progress of congenital sensory (cochlear) hearing loss varies and the inner ear may be malformed to different degrees. If this malformation involves both the cochlea and the vestibular organ, PDS is associated with vestibular areflexia with late motor milestones, delayed walking age, and balance problems. A characteristic feature of PDS is enlarged vestibular aqueducts [3]. Although PDS is one of the most frequent forms of syndromic deafness, with the estimated incidence of 1–9:100,000 worldwide [8], it has no distinct founder populations as seen for the *SLC26A3*-associated CLD.

While pathogenic *SLC26* variants are rare and cases mostly solitary, we report an unusual co-occurrence of two rare homozygous mutations in both the *SLC26A3* and *SLC26A4* genes, causing a rare combination of both CLD and PDS in two siblings. This finding uncovers the clinical challenges and genetic risk associated with recessive traits in the progeny of consanguineous families.

## Case presentation

The parents were healthy first cousins from Lebanon, currently living in Sweden. Furthermore, the parents of the mother were also first cousins. Family history involved no hearing disorders or gastrointestinal diseases. The mother had a history of five pregnancies and four deliveries. The first child was stillborn; a healthy boy was born 1 year thereafter, and the affected boy 1 year later. Three years later, the fourth pregnancy was complicated by an early miscarriage, and 1 year thereafter, the affected girl was born.

### The affected boy

Pregnancy ultrasound at gestational week 29 displayed polyhydramnios and dilated intestinal loops in the fetus, resulting in a suspicion of atresia. The boy was born at 36 weeks with a birth weight of 3075 g. He had a distended abdomen, but normal findings in colon radiology and small-bowel passage excluded intestinal atresia. Oral nutrition was started but the baby lost weight, the abdomen became more distended, and diarrhea was misinterpreted as urine. The boy deteriorated and developed hypokalemic metabolic alkalosis. Three weeks after the birth, a loop ileostomy was performed and a dilated intestine with poor peristalsis was noted. Four weeks later, a second operation was performed for a prolapse of the stoma, and two months after the birth, the stoma was removed due to a severe prolapse. Three days after the operation, a thrombosis in the brachiocephalic vein and the superior vena cava was detected and treated with dalteparin. Intestinal biopsies revealed normal histology, and metabolic tests as well as tests for cystic fibrosis were normal. Serum levels of electrolytes, bicarbonate and pH were normal when parenteral nutrition was given, but as soon as oral feeding was started, hypochloremic and hypokalemic metabolic alkalosis appeared. Two months after the birth, a suspicion of CLD was raised and confirmed by measuring high fecal chloride. Treatment with a solution of NaCl and KCl was started orally according to the recommendations [6]. Blood tests became normal and the abdomen became less distended but watery stools ten times a day or more persisted. After years of follow-up, the boy, currently 12 years old, still passes 7–8 watery stools per day but has normal growth and development without signs of hypochloremia or metabolic alkalosis. Treatment with the solution of NaCl and KCl is lifelong. Repeated tests for renal function, ultrasounds of the kidneys, electrolyte balance and thyroidal status have been normal.

The routine otoacoustic emission test (OAE) at birth was normal, but at the age of 4, a bilateral moderate sensory hearing impairment was diagnosed [a pure tone average (PTA4, 0.5, 1, 2, 4 kHz), right ear 89 dBHL, left ear 55 dBHL] using play audiometry. OAE was negative, and tympanometry showed normal curves. The hearing loss was suspected because of poor language acquisition and because the younger sister had just been diagnosed with the same condition. The hearing loss was progressive in the right ear but remained stable in the left. The boy received bilateral hearing aids and is now being considered for cochlear implants. Currently at the age of 12, he is attending a special school for hard of hearing/deaf children and communicates with spoken language as well as with some sign language.

### The affected girl

Pregnancy ultrasound at gestational week 25 showed a distended bowel but no polyhydramnios. A baby girl was born at 35 weeks with a birth weight of 2600 g. Her abdomen was slightly distended and the meconium watery. After admission to the neonatal intensive care unit, oral feeding was started, but the baby developed hypokalemia, hypochloremia, hyponatremia, and metabolic alkalosis, similar to her older brother. High fecal chloride confirmed the diagnosis of CLD. Six days after the birth, salt replacement therapy was started according to the recommendations [6]. Blood tests normalized and the baby became livelier, but she continued to pass loose stools. She was discharged from hospital 2 weeks after the birth. At the current age of 8 years, she passes 7–8 watery stools per day but shows normal growth and development. As for her brother, the treatment with salt solution is lifelong. Repeated tests for renal function, ultrasounds of the kidneys, electrolyte balance and thyroidal status have been normal.

The girl failed the otoacoustic emission test (OAE) at 7 days and again at 12 days. At the age of 6 weeks, brainstem response audiometry revealed a bilateral moderate to severe sensory hearing impairment (right ear 50 dBHL, left ear 50 dBHL). Bilateral hearing aids were fitted. The hearing loss was progressive, and at the age of 5 years, she received a cochlear implantation (CI) on the right ear. The pre-operative radiological examinations (CT and MRI) revealed minor malformations of the cochleas, and the semicircular canals were normal. The internal auditory canals were wide and the vestibular aqueducts were enlarged (EVA). At the time of the operation, the so-called “gusher” (increased perilymphatic pressure) appeared as a characteristic of Pendred syndrome. CI in the right ear and a hearing aid on the left resulted in significantly improved language acquisition and speech. The girl currently attends a special preschool for hard of hearing/deaf children and learns both Swedish spoken language and sign language.

### Genetic diagnoses

Genetic testing for CLD was performed at our research laboratory (Department of Medical Genetics, University of Helsinki, Finland) when the boy was 1 year old. *SLC26A3* exons were amplified using intronic primers as described [9]. The Arab founder mutation c.559G > T (p.G187X) on intron 5 [1] was excluded and no other exons or exon-intron boundaries showed pathogenic variations. However, PCR amplification of exon 1 was unsuccessful from the patient sample.

The suspected deletion around the non-coding exon 1 was further mapped by multiple PCR and sequencing reactions on both the patient sample and the parental samples. All PCR reactions were run in a volume of 25 μL involving 1 x PCR buffer, 1.5 mM MgCl_2,_ 200 μM of each dNTPs, 0.8 μM of each primer, 0.03 U/μL of Hot Star Taq Plus DNA polymerase (Qiagen, Valencia, CA), and 10 ng of genomic DNA. Cycling conditions were 95 °C for 5 min, followed by 30 cycles of 95 °C for 30 s, 57 °C for 30 s, and 72 °C for 45 s, and by the final extension of 72 °C for 10 min. The PCR products were studied on 2% agarose gels. ExoSAP-IT PCR clean-up was performed according to manufacturer’s instructions (Thermo Fisher Scientific) and PCR products were further studied by direct sequencing into both directions utilizing the BigDye Terminator v.3.1 Cycle Sequencing Kit and the ABI3730xl DNA Analyzer (Thermo Fisher Scientific).

We designed primers to amplify flanking sequences around 16 single nucleotide polymorphisms (SNPs) from 54 kilobases upstream (rs10274491) to 5 kb downstream (rs4730264) from the exon 1 of the *SLC26A3* gene. We identified regions in which PCR was unsuccessful in the patient sample but successful in the parental samples. This approach allowed localization of the approximate borders of the deletion, which was further characterized using specific primers (5′-CCGTGTTCAAGGACAATGTG-3′ in intron 1 and 5′-TGAGCCAGTCAGTGCAAAAC-3′ in the promoter). The deletion involved 8596 nucleotides from 4.3 kilobases upstream of exon 1 to 4.2 kilobases downstream of it. This homozygous deletion found in the patient sample, and present in a heterozygous form in the parental samples, results in a deletion of a large segment including the intestinal promoter for transcription and the non-coding exon 1 of the *SLC26A3* gene [10]. Taking into account the typical clinical picture of CLD in the patient, this large deletion was implicated as the causative variation for CLD. When the sister was born, no further genetic test for *SLC26A3* was done because the clinical diagnosis of CLD – both the clinical picture and high fecal chloride – were consistent with the disease.

When hearing impairment was clinically diagnosed, both the boy and the girl had additional genetic testing (Department of Clinical Genetics/Rigshospitalet, Copenhagen, Denmark) and were found to be homozygous for the *SLC26A4* change c.384delT (p.Phe128Leufs#17). The parents were heterozygous carriers for this pathogenic variant. This deletion is previously unreported and not found in genetic databases but lies at the location where one pathogenic variant c.382_384delTTTinsAA (p.Phe128Lysfs) has been reported in a single individual [11].

**Fig. 1:**
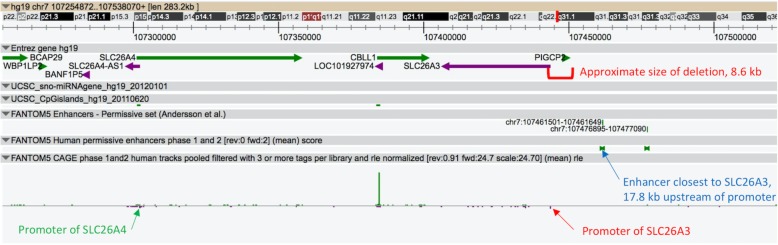
*SLC26A3* (purple arrow) and *SLC26A4* (green arrow) genes on chromosome 7q22.3-q31.1 (marked with red bar) are shown. The promoters of these genes are marked with arrows and the *SLC26A3* deletion causative for CLD with a red square bracket. The two nearest enhancer elements are shown, of which one is 17.8 kb upstream of *SLC26A3*; none is near *SLC26A4*. The 8.6 kb deletion responsible for CLD extends upstream only about half-way to the nearest enhancer element. The image is a screen capture from the FANTOM5 database (http://fantom.gsc.riken.jp/zenbu)

The region covering *SLC26A3* and *SLC26A4* genes, including their promoters, is shown in Fig. 1. These genes are located in opposite strands on 7q22.3-q31.1 and the *SLC26A3* deletion found in the patient does not cover any enhancer elements of the gene. Thereby, the co-occurrence of these diseases is apparently not affected by any enhancers, further supporting the causative role of the two rare and independent mutations for CLD and PDS in these patients.

## Discussion and conclusions

The described co-occurrence of two rare recessive pathogenic variants independent of each other is highly unusual and previously unreported for the members of the *SLC26* gene family. As for CLD, founder populations in Finland, Poland and the Arab Middle East account for the majority of the affected individuals [1]. The affected siblings described here are, however, homozygous for a large deletion which is solitary but enriched in the progeny for consanguinity in the family. No other such large deletions have been described in association with CLD [1]. As this deletion of 8.6 kb is still distantly located (50 kb) from the *SLC26A4* gene and not covering any enhancer elements of the genes (Fig. 1), the rare *SLC26A4* nonsense change is most likely independently causative for PDS in these patients. These cases highlight the importance of consanguineous family history in diagnostics of rare childhood disorders.

The clinical picture of CLD in these patients was typical as such, and the age at the diagnosis of the older child in the family was close to the mean of 2.7 months reported in the literature [7]. While evident genotype-phenotype correlation in CLD remains non-existent and functional studies of different *SLC26A3* disease mutations have revealed consistent loss of function [12], differential clinical presentation in these siblings supports earlier conclusions that modifier genes may affect the clinical picture of CLD [13]. This is supported by the differential severity of diarrhea in these siblings in the infancy. Additionally, the younger child showed fewer ultrasonic features of CLD while in utero, although the characteristics of CLD include intrauterine onset of watery diarrhea, polyhydramnios, and dilated intestinal loops in the fetus [5]. Notably, unnecessary operations before the diagnosis of CLD in the older sibling may have modulated his diarrheal symptoms. The deep venous thrombosis in the boy might be associated with severe dehydration, as no thromboembolic complications have arisen in larger series of CLD [7]. Because chronic dehydration reduces the volume of diarrhea, diagnostics of CLD is challenging and often leads to suspicion of intestinal obstruction [7]. However, once the disorder is suspected, the diagnosis is easily made by measuring fecal chloride [6], as also described here. Salt replacement therapy allows normal growth and development in a patient with CLD [7], as seen in both the siblings described here.

Both the boy and the girl showed a typical pattern of hearing loss attributed to Pendred syndrome. This hearing loss is often progressive and, in many cases, profound in young adulthood [3]. The radiological findings of enlarged vestibular aqueduct (EVA) and an enlarged meatus acusticus internus is typical for PDS, as is the gusher found during the girl’s operation when opening the round window. These siblings have not so far shown signs of hypothyroidism, which may be absent in cases with late-onset PDS [14].

SLC26 anion transporters show a highly differential tissue distribution [12]. *SLC26A3* is mostly expressed by the apical intestinal epithelium and the male reproductive tract [15], while *SLC26A4* shows expression in the inner ear and the thyrocyte [16]. Although loss of function of these two SLC26 anion transporters is unlikely to have any additive effects, their possible consequences are related to kidney function. Pendrin shows expression on the apical membrane of the renal collecting duct intercalated cell [17]. While loss of *SLC26A4* alone is compensated with other transporters in the nephron, and neither Pendred patients nor *SLC26A4*-deficient mice develop overt acid-base disturbances [18], *SLC26A4* in the kidneys is regulated by systemic chloride load and acid-base status [19]. Furthermore, untreated CLD is associated with hypochloremic and hypokalemic metabolic alkalosis and a risk for renal calcifications and chronic kidney disease [20]. Therefore, the life-long chloride-losing diarrhea of CLD together with the loss of function of renal SLC26A4-mediated transport might modulate the risk of kidney disease in these siblings. Their co-occurrence of CLD and PDS thereby underlines the importance of salt replacement therapy and maintenance of normal acid-base and electrolyte balance.

This study describes an unusual presentation of two unique recessive diseases caused by homozygous changes in two nearby genes, *SLC26A3* and *SLC26A4*. The clinical picture in the siblings highlights the challenges in diagnostics of rare disorders in children of consanguineous couples.

## Data Availability

The datasets generated and/or analyzed during the current study are not publicly available because it is possible that individual privacy could be compromised.

## Abbreviations

CLD: Congenital chloride diarrhea
*SLC26*: Solute carrier family 26
PDS: Pendred syndrome
OAE: Otoacoustic emission test
dBHL: Decibel hearing level
EVA: Enlarged vestibular aqueduct
CI: Cochlear implantation
